# Benchmark Ab Initio Mapping of the F^–^ + CH_2_ClI S_N_2 and Proton-Abstraction Reactions

**DOI:** 10.1021/acs.jpca.4c06716

**Published:** 2024-12-02

**Authors:** Domonkos A. Tasi, Erik M. Orján, Gábor Czakó

**Affiliations:** MTA-SZTE Lendület “Momentum” Computational Reaction Dynamics Research Group, Interdisciplinary Excellence Centre and Department of Physical Chemistry and Materials Science, Institute of Chemistry, University of Szeged, Szeged H-6720, Hungary

## Abstract

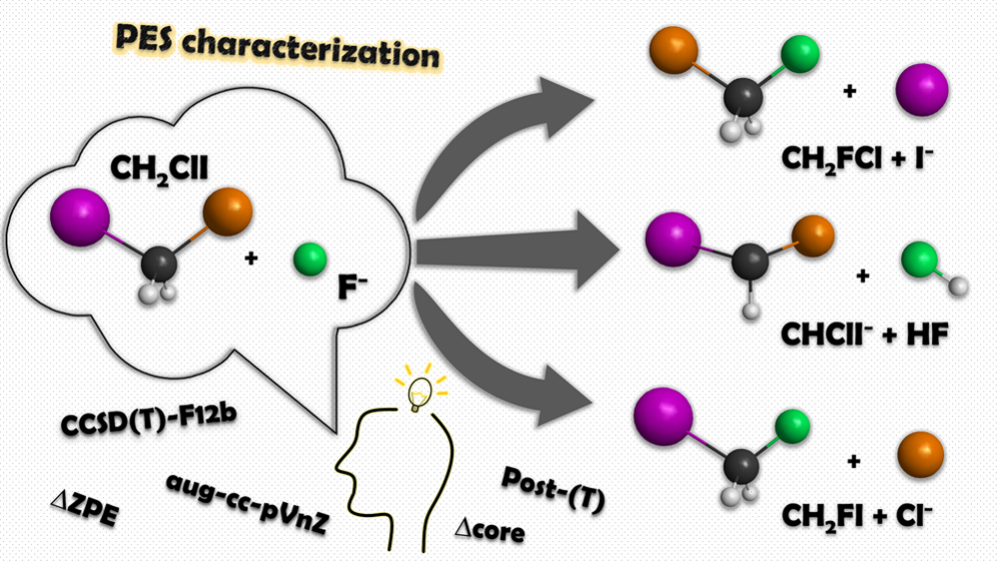

The experimental and theoretical studies of gas-phase S_N_2 reactions have significantly broadened our understanding of the mechanisms governing even the simplest chemical processes. These investigations have not only advanced our knowledge of reaction pathways but also provided critical insights into the fundamental dynamics of chemical systems. Nevertheless, in the case of the prototypical X^–^ + CH_3_Y → Y^–^ + CH_3_X [X, Y = F, Cl, Br, and I] S_N_2 reactions, the effect of the additional halogenation of CH_3_Y has not been thoroughly explored. Thus, here, we perform the first high-level ab initio characterization of the F^–^ + CH_2_ClI S_N_2 and proton-abstraction reactions utilizing the explicitly-correlated CCSD(T)-F12b method. Two possible S_N_2 channels leading to the Cl^–^ + CH_2_FI and I^–^ + CH_2_FCl products are distinguished, in which we investigate four different pathways of back-side attack Walden inversion, front-side attack, double inversion, and halogen-bonded complex formation. In order to obtain the benchmark energies of the geometries of the stationary points, determined at the CCSD(T)-F12b/aug-cc-pVTZ level of theory, additional computations are carried out considering the basis set effects, post-CCSD(T) correlations, and core corrections. Using the benchmark data, we assess the accuracy of the MP2, DF-MP2, MP2-F12, and DF-MP2-F12 methods as well. By comparing the present F^–^ + CH_2_ClI system with the corresponding F^–^ + CH_3_Y [Y = Cl and I] reactions, this study demonstrates that further halogenation of CH_3_Y significantly promotes the corresponding proton-abstraction and S_N_2 retention channels as well as the halogen-bonded complex formation, and as a consequence, the traditional back-side attack Walden-inversion mechanism becomes less pronounced.

## 1 Introduction

The advancements in our comprehension of the fundamental bimolecular nucleophilic substitution (S_N_2) reactions have substantially progressed over the last few decades.^1−6^ The development of the crossed-beam velocity map imaging technique for ion–molecule reactions enabled us to study S_N_2 reactions in detail;^7,8^ however, utilizing theoretical methods, such as the quasi-classical trajectory (QCT) approach, remained also crucial.^9−11^ With these methods at hand, the complexity of the dynamics of elemental X^–^ + CH_3_Y → Y^–^ + CH_3_X [X, Y = F, Cl, Br and I] S_N_2 reactions has been unveiled, leading to the identification of several possible indirect and direct channels.^12−16^ For the Cl^–^ + CH_3_I S_N_2 reaction, a roundabout mechanism was revealed by Hase and co-workers,^7^ while for F^–^ + CH_3_Cl, our QCT simulations uncovered a novel indirect low-energy retention route, the so-called double-inversion pathway.^17^ In the case of F^–^ + CH_3_I, the formation of a H-bonded F^–^···HCH_2_I complex was found to exert a substantial influence on the S_N_2 mechanism.^18^ A comparative study on the F^–^ + CH_3_Cl/CH_3_I S_N_2 reactions was reported in order to examine the effect of the leaving Cl/I groups on the dynamics.^19^ In 2023, a global analytical ab initio potential energy surface was developed for Cl^–^ + CH_3_I to describe the dynamics of the reaction at a wide range of collision energy.^20^ Moreover, the microsolvated X^–^(H_2_O)_*n*_ + CH_3_I [X = F, Cl and *n* = 1 – 3] S_N_2 reactions were investigated experimentally and theoretically as well.^21−28^ It is noteworthy that the stationary points of the abovementioned S_N_2 reactions have also been characterized in several papers.^29−34^

On one hand, to enhance the complexity of the X^–^ + CH_3_Y S_N_2 reactions, one can substitute methyl halides with different ethyl halides.^6^ In these cases, bimolecular elimination (E2) can also take place competing with S_N_2.^35−43^ On the other hand, the impact of the halogenation on the reactions involving methyl and ethyl halides can also be considered.^6,11^ However, research is somewhat lacking on that matter; a small number of studies were conducted on the corresponding S_N_2 and E2 reactions.^44−54^ Hine and co-workers showed that additional halogenation of methyl halides results in a proportional decrease in S_N_2 reactivity, correlating with the weights of the halogenes.^55^ Cardini and co-workers compared the Cl^–^ + XCH_2_Cl → XCH_2_Cl + Cl^–^ [X = H, Cl, and CN] identity S_N_2 reactions by performing ab initio molecular dynamics calculations and revealed the formation of a H-bond between the reactants in the entrance channel.^51,56^ A series of S_N_2 reactions of halide ions and trifluoromethyl halides were analyzed by Bogdanov and McMahon concentrating on the ion–molecule complexes and transition states of the back- and front-side attack pathways, as well as of other possible mechanisms.^52^ Regarding the [I···ICF_3_]^−^ front-side complex of the I^–^ + CF_3_I S_N_2 reaction, Verlet and co-workers reported a detailed study utilizing photoelectron spectroscopy and density functional theory.^54^ Apart from the abovementioned papers, it should be also noted that reactions involving various fluorinated methyl halides were the main focus of other examinations.^49,57−59^ Recently, the groups of Wester and Viggiano, in collaboration with our group, compared the F^–^ + CH_3_CH_2_I and F^–^ + CF_3_CH_2_I reactions to assess the influence of fluorination of the β-carbon center on the dynamics.^46^

Thus, in the present study, we report the first thorough theoretical study on the potential energy surface of the F^–^ + CH_2_ClI reaction using the high-level explicitly-correlated CCSD(T)-F12b method. Two distinct S_N_2 channels resulting in the products of Cl^–^ + CH_2_FI and I^–^ + CH_2_FCl are considered; moreover, the competing proton-abstraction pathway leading to HF + CHClI^–^ is also characterized. In the case of the S_N_2 paths, several mechanisms are investigated: back-side attack, front-side attack, double inversion,^17^ and halogen-bonded complex formation.^34,60^ In addition, with the benchmark values in hand, we also aim to evaluate the performance of alternative lower-level ab initio methods. The current work initiates a detailed investigation of the title reaction, which is a key step toward developing a global analytical potential energy surface in order to analyze the reaction dynamics using QCT simulations. Experimental studies of the title reaction are also being conducted by the Wester group using the crossed beam ion-imaging technique. Theoretical approaches are crucial in complementing these experiments; as demonstrated in prior studies,^61,62^ they yield insights beyond the reach of experimental methods. In Section 2, the computational details are described, and in Section 3, the results are presented and discussed. Finally, a summary and conclusions are provided in Section 4.

## 2 Computational Details

The stationary points of the potential energy surfaces of the F^–^ + CH_2_ClI S_N_2 and proton-abstraction channels are explored by using the second-order Møller–Plesset perturbation theory (MP2) method with the augmented correlation-consistent polarized-valence-double-ζ (aug-cc-pVDZ) basis set.^63,64^ Afterward, the explicitly-correlated coupled cluster singles, doubles, and perturbative triples CCSD(T)-F12b method with the aug-cc-pV*n*Z [*n* = 2 and 3] basis sets is applied to determine the structures, energies, and harmonic vibration frequencies of the stationary points.^65^ The benchmark energies are computed at the CCSD(T)-F12b/aug-cc-pVTZ structures employing the CCSD(T)-F12b method with the aug-cc-pVQZ basis set, as well as considering (a) post-CCSD(T) effects and (b) core-correlation corrections, where single-point energy computations of (a) CCSD(T),^66^ CCSDT,^67^ and CCSDT(Q)^68^ with the aug-cc-pVDZ basis set and (b) CCSD(T)/aug-cc-pwCVTZ with both frozen-core (FC) and all-electron (AE) approaches are performed.^69^ Small-core relativistic effective core potential is employed for I,^70^ and the aug-cc-pV*n*Z-PP [*n* = 2–4] and aug-cc-pwCVTZ-PP basis sets are applied to replace the inner-core 1s^2^ 2s^2^ 2p^6^ 3s^2^ 3p^6^ 3d^10^ electrons. At the MP2/aug-cc-pVDZ level of theory, intrinsic reaction coordinate computations are also performed from the saddle points in order to provide a more in-depth characterization of the title reaction.

Hence, the benchmark classical (adiabatic) relative energies of the stationary points are obtained as
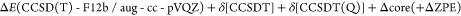
1where
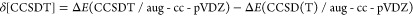
2
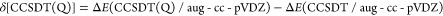
3
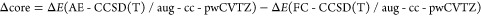
4and ΔZPE is the harmonic zero-point energy correction obtained at the CCSD(T)-F12b/aug-cc-pVTZ level of theory. It is important to note that in the case of the classical energy, the nuclei are fixed at the corresponding stationary point during the calculations, meaning the energy is classical from the perspective of the nuclei, while for the adiabatic energy, it is assumed that the nuclei are in their ground vibrational state, as defined by the principles of quantum mechanics. Thus, in the case of the classical energy, the ZPE correction is not included, whereas for the adiabatic energy, which is typically presented in parentheses after the classical energy, ZPE is included. The post-CCSD(T) correction is obtained from the sum of eqs 2 and 3; however, in the present work, we examined them separately to assess the impact of each term. It should also be emphasized that, based on eqs 2 and 3, no convergence is assumed for the post-CCSD(T) corrections since the corresponding δ[CCSDT] is obtained relative to the CCSD(T)/aug-cc-pVDZ energy, not the CCSD/aug-cc-pVDZ energy.

In the course of the assessment of the alternative MP2 methods,^71,72^ the geometries and energies of the stationary points are also determined using the density-fitted DF-MP2, explicitly-correlated MP2-F12, and the corresponding DF-MP2-F12 methods with aug-cc-pV*n*Z [*n* = 2 and 3]. All the ab initio computations are carried out with the Molpro program package,^73^ and for CCSDT and CCSDT(Q), the MRCC program interfaced to Molpro is employed.^74,75^

## 3 Results and Discussion

**Figure 1:**
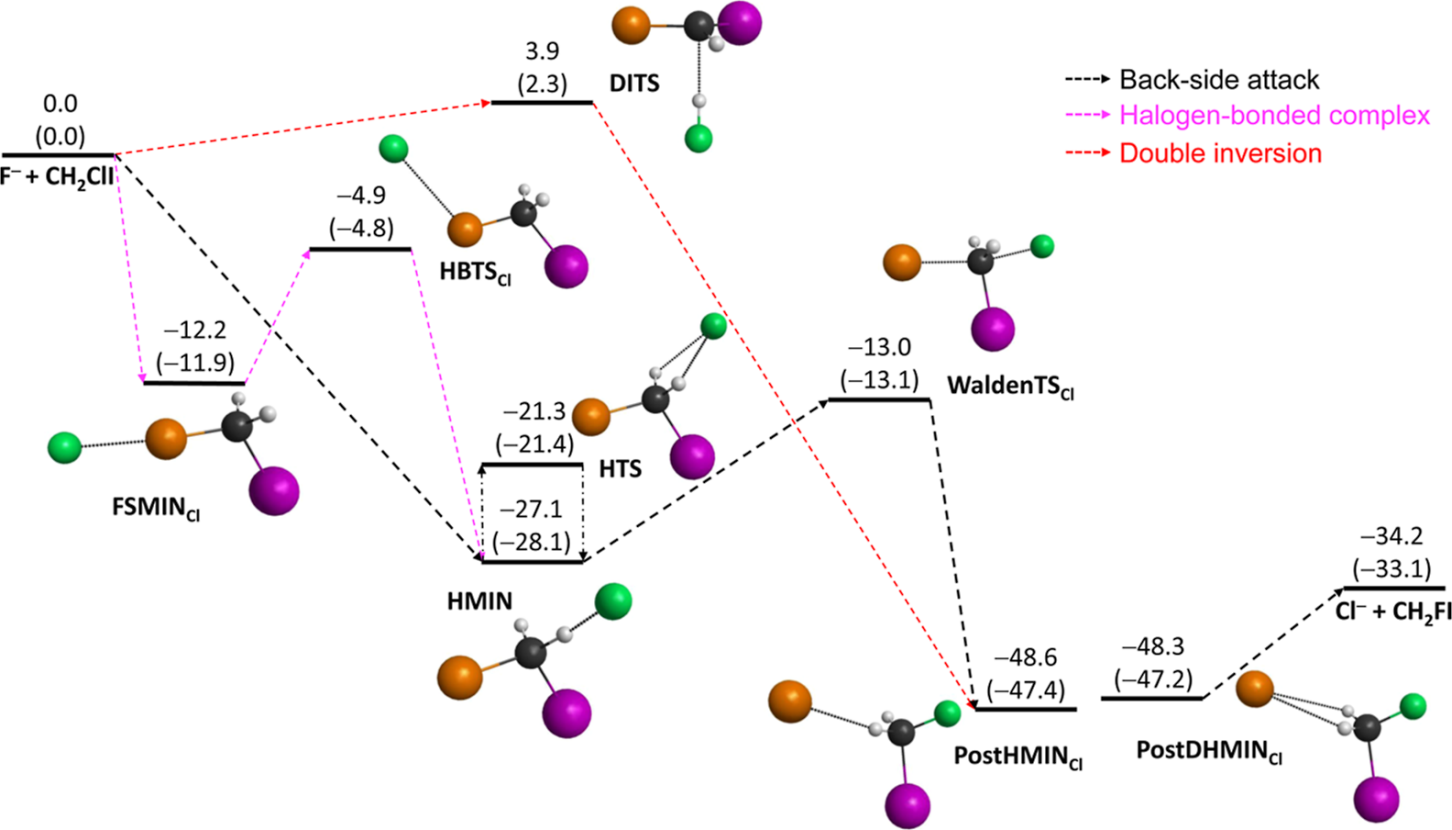
Schematic potential energy surface of the F^–^ + CH_2_ClI → Cl^–^ + CH_2_FI S_N_2 reaction showing the benchmark classical (adiabatic) relative energies (kcal/mol) of the stationary points along the possible pathways. Color scheme: carbon—black, iodine—purple, chlorine—orange, fluorine—green, and hydrogen—gray.

**Figure 2:**
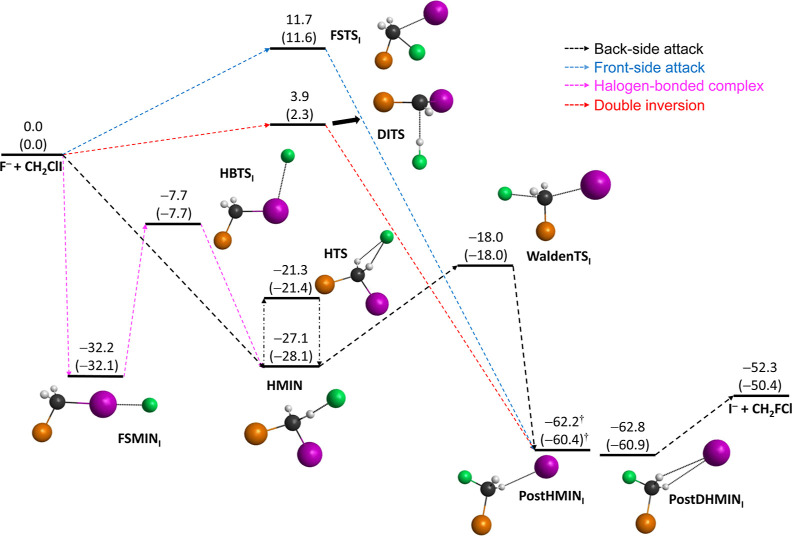
Schematic potential energy surface of the F^–^ + CH_2_ClI → I^–^ + CH_2_FCl S_N_2 reaction showing the benchmark classical (adiabatic) relative energies (kcal/mol) of the stationary points along the possible pathways. Results noted with † correspond to the MP2/aug-cc-pVDZ structure. Color scheme: carbon—black, iodine—purple, chlorine—orange, fluorine—green, and hydrogen—gray.

**Figure 3:**
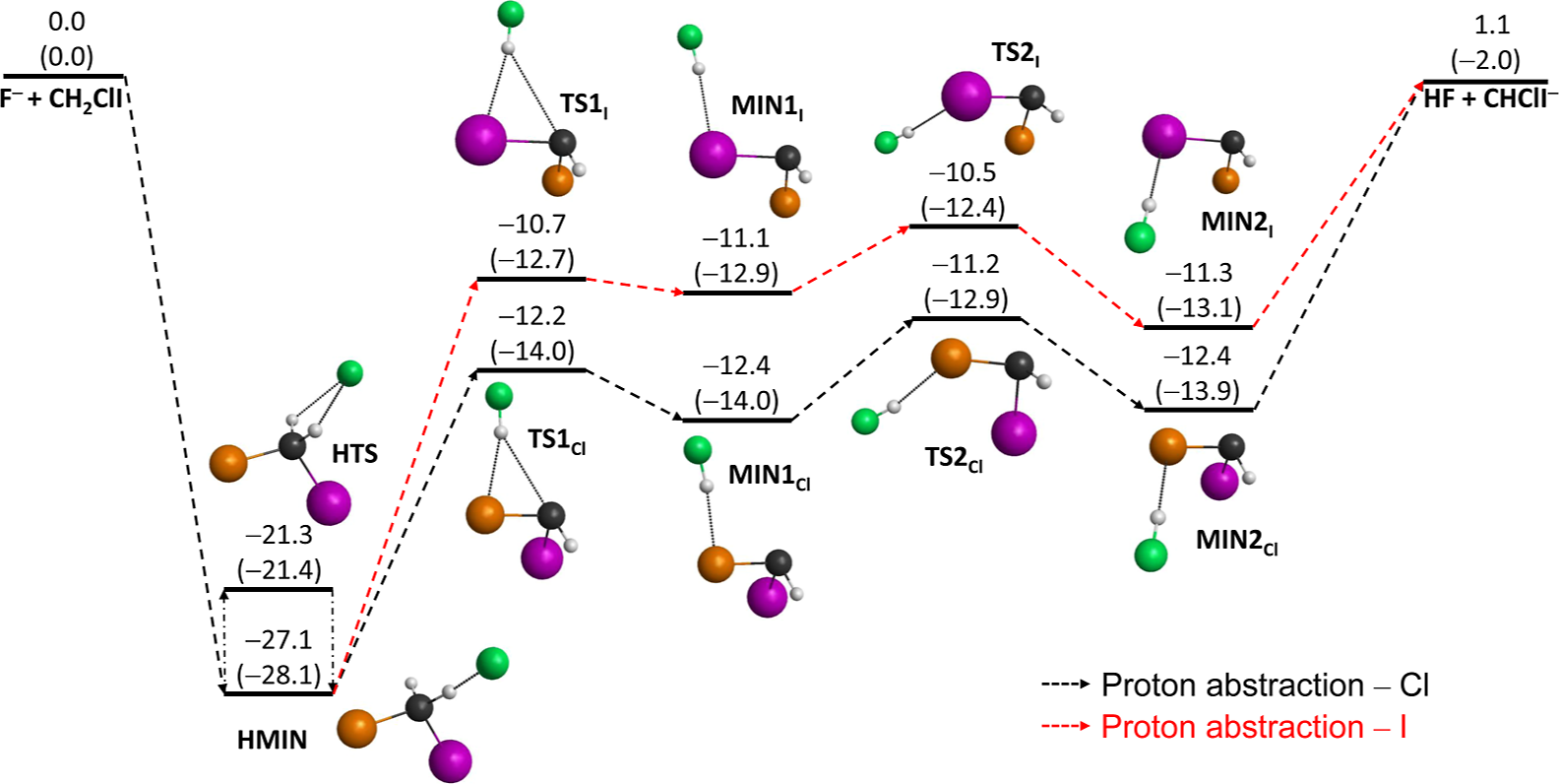
Schematic potential energy surface of the F^–^ + CH_2_ClI → HF + CHClI^–^ proton-abstraction reaction showing the benchmark classical (adiabatic) relative energies (kcal/mol) of the stationary points along the possible pathways. Color scheme: carbon—black, iodine—purple, chlorine—orange, fluorine—green, and hydrogen—gray.

**Figure 4:**
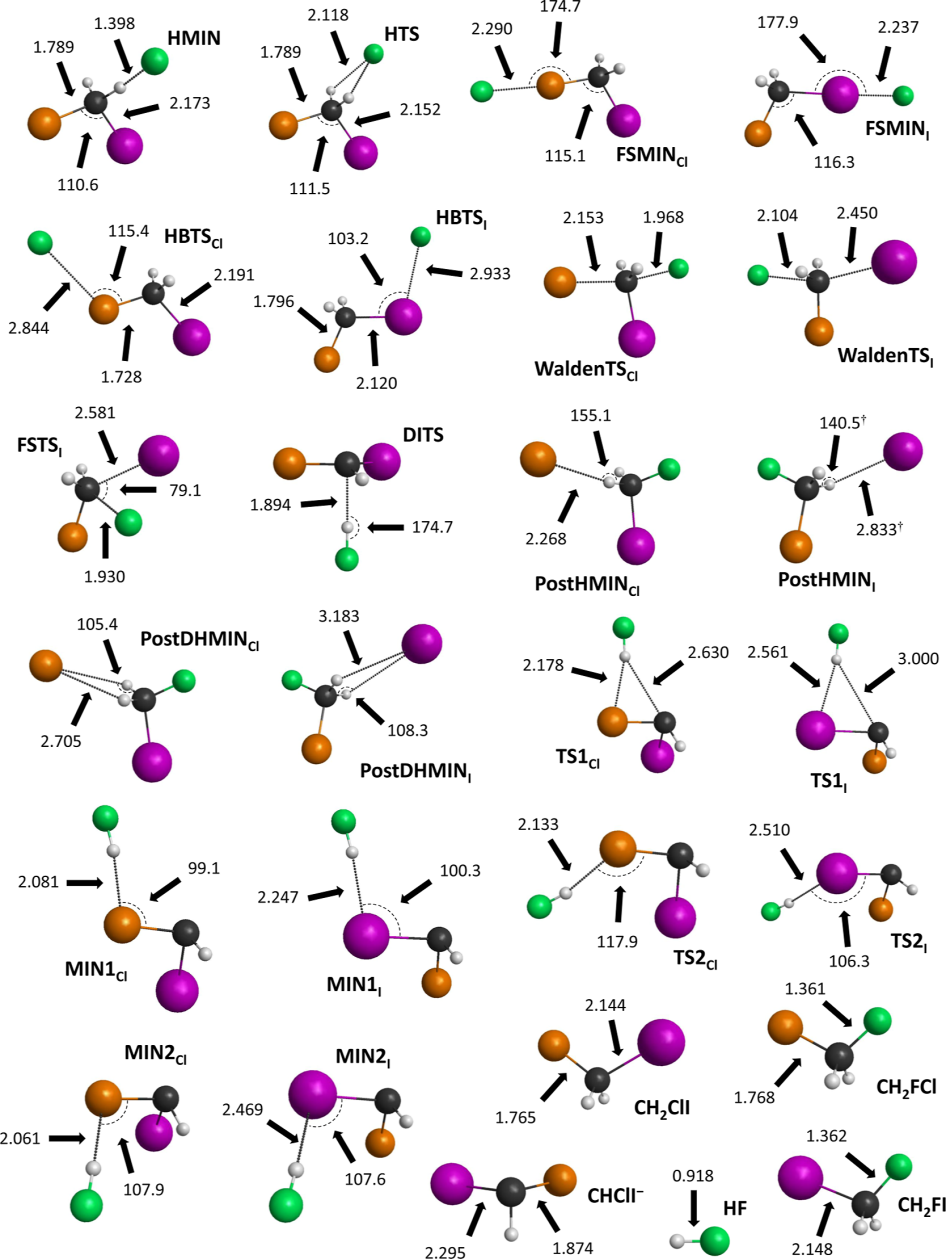
Most important bond lengths (Å) and angles (°) of the stationary points of the F^–^ + CH_2_ClI S_N_2 and proton-abstraction reactions obtained at the CCSD(T)-F12b/aug-cc-pVTZ level of theory. Results noted with † are obtained with MP2/aug-cc-pVDZ. Color scheme: carbon—black, iodine—purple, chlorine—orange, fluorine—green, and hydrogen—gray.

In the case of the F^–^ + CH_2_ClI reaction, the schematic potential energy surfaces of the two possible S_N_2 channels as well as of the proton-abstraction path featuring the benchmark classical (adiabatic) relative energies of the stationary points along the possible routes are presented in Figures 1–3, respectively. Note that the corresponding stationary points are marked with “Cl” or “I”, depending on the associated S_N_2 channels of Cl^–^ + CH_2_FI or I^–^ + CH_2_FCl or on the viable proton-abstraction pathways. The geometries of the stationary points are depicted in Figure 4, showing the most important structural parameters. The most accurate CCSD(T)-F12b/aug-cc-pVTZ Cartesian coordinates of the complexes, transition states, reactants, and products are provided in the Supporting Information. The relative energies obtained at different ab initio levels of theory, together with the post-CCSD(T), core, relativistic, and ZPE corrections, are given in Table 1.

**Table 1:** Benchmark Classical and Adiabatic Relative Energies (kcal/mol) of the Stationary Points, Relative to the F^–^ + CH_2_ClI Reactants, for the S_N_2 and Proton-Abstraction Channels of the F^–^ + CH_2_ClI Reaction

F^–^ + CH_2_ClI	DZ^a^	TZ^b^	QZ^c^	δT^d^	δ(*Q*)^e^	Δcore^f^	classical^g^	ΔZPE^h^	adiabatic^i^
HMIN	–27.56	–27.33	–27.17	–0.02	–0.02	0.06	–27.14	–0.96	–28.11
HTS	–21.65	–21.43	–21.27	–0.04	–0.01	0.02	–21.31	–0.08	–21.38
FSMIN_Cl_	–12.30	–12.22	–12.08	–0.02	–0.14	0.04	–12.20	0.31	–11.89
FSMIN_I_	–32.33	–32.86	–32.82	0.05	–0.16	0.68	–32.24	0.19	–32.06
HBTS_Cl_	–5.06	–4.86	–4.78	–0.05	–0.04	–0.03	–4.90	0.07	–4.83
HBTS_I_	–7.25	–7.68	–7.64	–0.04	–0.03	–0.03	–7.74	0.03	–7.71
WaldenTS_Cl_	–12.65	–13.17	–13.09	–0.08	–0.19	0.36	–12.99	–0.13	–13.12
WaldenTS_I_	–18.14	–18.07	–18.00	–0.06	–0.23	0.25	–18.03	0.00	–18.03
FSTS_I_	11.54	11.58	11.73	–0.03	–0.53	0.50	11.67	–0.08	11.58
DITS	3.60	3.66	3.76	0.11	–0.14	0.19	3.92	–1.63	2.29
PostHMIN_Cl_	–48.77	–48.81	–48.82	–0.03	0.13	0.15	–48.56	1.17	–47.39
PostHMIN_I_	–63.09	–62.86^j^	–63.14^j^	–0.01^j^	0.20^j^	0.74^j^	–62.21^j^	1.84^j^	–60.38^j^
PostDHMIN_Cl_	–48.46	–48.55	–48.60	–0.02	0.14	0.17	–48.32	1.08	–47.23
PostDHMIN_I_	–63.61	–63.40	–63.74	0.01	0.22	0.67	–62.84	1.91	–60.93
MIN1_Cl_	–12.47	–12.90	–12.92	–0.05	–0.10	0.64	–12.43	–1.59	–14.03
MIN1_I_	–10.97	–11.48	–11.41	–0.07	–0.10	0.47	–11.11	–1.79	–12.90
TS1_Cl_	–12.33	–12.66	–12.67	–0.05	–0.10	0.61	–12.21	–1.81	–14.02
TS1_I_	–10.61	–11.09	–11.03	–0.07	–0.10	0.46	–10.73	–1.99	–12.72
MIN2_Cl_	–12.34	–12.83	–12.85	–0.05	–0.11	0.64	–12.38	–1.50	–13.88
MIN2_I_	–11.11	–11.63	–11.56	–0.07	–0.10	0.46	–11.27	–1.79	–13.06
TS2_Cl_	–11.18	–11.68	–11.71	–0.05	–0.11	0.62	–11.25	–1.67	–12.92
TS2_I_	–10.40	–10.90	–10.84	–0.07	–0.10	0.48	–10.53	–1.84	–12.37
Cl^–^ + CH_2_FI	–34.23	–34.48	–34.55	–0.00	0.18	0.23	–34.15	1.03	–33.12
I^–^ + CH_2_FCl	–53.22	–53.03	–53.46	0.03	0.24	0.92	–52.27	1.84	–50.43
HF + CHClI^–^	1.39	0.75	0.54	–0.06	–0.09	0.74	1.13	–3.15	–2.01

a CCSD(T)-F12b/aug-cc-pVDZ.

b CCSD(T)-F12b/aug-cc-pVTZ.

c CCSD(T)-F12b/aug-cc-pVQZ relative energies at the CCSD(T)-F12b/aug-cc-pVTZ geometries.

d CCSDT/aug-cc-pVDZ – CCSD(T)/aug-cc-pVDZ.

e CCSDT(Q)/aug-cc-pVDZ – CCSDT/aug-cc-pVDZ.

f AE-CCSD(T)/aug-cc-pwCVTZ – FC-CCSD(T)/aug-cc-pwCVTZ.

g QZ + δT + δ(Q) + Δcore.

h ΔZPE(CCSD(T)-F12b/aug-cc-pVTZ).

i QZ + δT + δ(Q) + Δcore + ΔZPE.

j MP2/aug-cc-pVDZ geometry and frequencies.

As shown in Figures 1 and 2, both S_N_2 channels are submerged, and since iodine is the best leaving group among the halogens, the formation of I^–^ + CH_2_FCl is more exothermic than that of the Cl^–^ + CH_2_FI products by 18.1 (17.3) kcal/mol. In the entrance region of the S_N_2 channels, the same stationary points of HMIN and HTS (the H-bonded minimum and H-bonded transition state) are located. Considering the formerly characterized halogen-bonded (front-side) complex formation,^34,60^ FSMIN_I_ is situated below FSMIN_Cl_ by 20.0 (20.2) kcal/mol; however, for the halogen-bonded transition states (HBTS_Cl_ and HBTS_I_), the energy difference (<3 kcal/mol) is not that significant. As seen in Figure 4, only minor differences in the structural parameters of HBTS_Cl_ and HBTS_I_ can be seen, except for the F^–^···Cl–C and F^–^···I–C bond angles, differing from each other by ∼12°. It should also be highlighted that FSMIN_I_ is the most stable complex in the reactant region, and no traditional F^–^···H_2_CClI ion–dipole prereaction complex can be identified. In the case of the I^–^ + CH_2_FCl S_N_2 channel, the barrier height of the back-side attack mechanism is lowered by 5.0 (4.9) kcal/mol compared to the Cl^–^ + CH_2_FI channel; thus, based on the energetics of the reaction, the formation of the I^–^ + CH_2_FCl products is more favored kinetically as well as thermodynamically. The C–Cl bond at WaldenTS_Cl_ is stretched by 0.388 Å and the C–I bond at WaldenTS_I_ is lengthened by 0.306 Å, relative to the corresponding bond lengths in the CH_2_ClI reactant. In the case of the C–F bonds at WaldenTS_Cl_ and WaldenTS_I_, an increase of 0.607 and 0.742 Å can be found, relative to the products of CH_2_FCl and CH_2_FI, in order. The global minimum of each S_N_2 channel is found in the exit region with relative energies of −48.6 (−47.4) and −62.8 (−60.9) kcal/mol at the single and double H-bonded postreaction minima (PostHMIN_Cl_ and PostDHMIN_I_), respectively. Similar to the postreaction complexes of the X^–^ + CH_3_Y → Y^–^ + CH_3_X [Y = F, Cl, Br, and I; X = OH, NH_2_, PH_2_, OOH, etc.] reactions,^76−78^ the dissociation energies of the leaving Cl^–^ at PostHMINs and PostDHMINs are larger than in the cases of I^–^, and the H···Cl^–^ bonds are shortened by 0.5–0.6 Å relative to the corresponding H···I^–^ bonds. Noteworthily, based on the determined harmonic vibrational frequencies of the intermolecular modes, it is uncertain whether the PostDHMIN_Cl_ and PostDHMIN_I_ are minima or transition states. In this study, the structures identified in the product region are treated as minima. The PostHMIN_I_ complex cannot be considered stable with complete certainty as it can be determined only at the MP2/aug-cc-pVDZ and DF-MP2/aug-cc-pVDZ levels of theory. At other levels of theory, geometry optimization results in the PostDHMIN_I_ structure. However, in order to present a thorough characterization of the title reaction, the corresponding PostHMIN_I_ complex is also included in our examination. Concerning the symmetries of the aforementioned stationary points, the Cl and I variants of FSMIN, HBTS, HTS, WaldenTS, and PostDHMIN have *C*_s_ symmetry.

In a simple X^–^ + CH_3_Y S_N_2 reaction, retention in the initial configuration can occur via two distinct pathways: front-side attack and double inversion. The front-side attack substitution is a direct mechanism, which goes through a high-energy transition state of [XYCH_3_]^−^. In contrast, double inversion begins with a proton abstraction by the F^–^, and the system lacks sufficient energy to dissociate; therefore, HF circulates around CH_2_Cl^–^, subsequently forming a C–H bond and the configuration around the carbon center gets inverted. Afterward, a traditional Walden inversion takes place, which involves the formation of a C–F bond and the breaking of the C–Cl bond, resulting in retention of the initial configuration through an indirect mechanism.^17^ In the case of the I^–^ + CH_2_FCl channel, the transition state (FSTS_I_) can be identified for the front-side attack pathway with a relative energy of 11.7 (11.6) kcal/mol; however, in the alternative Cl^–^ + CH_2_FI S_N_2 channel, the corresponding FSTS_Cl_ cannot be determined. Relying on the structural considerations of the reactants, it can be concluded that the structure of FSTS_Cl_ should be similar or even identical to that of FSTS_I_, and differences in the lengths of the C–Cl and C–I bonds may occur; however, further theoretical and experimental investigations are necessary to validate these assumptions. The barrier of double inversion is only 3.9 (2.3) kcal/mol, and the DITS is below FSTS_I_ by 7.7 (9.3) kcal/mol.

The schematic representation of the proton-abstraction channel of the F^–^ + CH_2_ClI reaction is depicted in Figure 3. Based on the stationary-point characterization, two different reaction pathways are proposed for proton abstraction depending on the departure of the HF product. Analogous to S_N_2, each stationary point of proton abstraction is below the reactant asymptote, and with considering the ZPE corrections, the reaction turns out to be exothermic with an enthalpy of −2.0 kcal/mol. The proposed pathways are energetically distinct from each other as the Cl-labeled stationary points are located deeper than the I-labeled ones by more than 1 kcal/mol in each case. However, it is important to note that these two reaction pathways should not be considered completely separate as overlap between them may take place during the proton-abstraction mechanism.

In comparison to the F^–^ + CH_3_Y [Y = Cl and I] S_N_2 reactions, the replacement of a H in CH_3_Y with either a Cl or I significantly changes the energetics of the potential energy surfaces.^17,32^ The S_N_2 channels (Cl^–^ + CH_2_FI and I^–^ + CH_2_FCl) of the title reaction are more exothermic than the corresponding F^–^ + CH_3_Y → CH_3_F + Y^–^ [Y = Cl and I] reactions, submerged by more than 2 (Cl) and 5 (I) kcal/mol. However, the formation of the Cl^–^ + CH_2_FI and I^–^ + CH_2_FCl products is less preferred kinetically as the classical barriers of WaldenTS_Cl_ (14.1 kcal/mol) and WaldenTS_I_ (9.1 kcal/mol) relative to HMIN are remarkably higher than those of the transition states of [F···CH_3_···Cl]^−^ (3.4 kcal/mol) and [F···CH_3_···I]^−^ (0.2 kcal/mol). In the cases of the S_N_2 retention pathways, a different situation emerges: The classical relative energy of DITS is lower than that of the transition states of the F^–^ + CH_3_Y reactions by more than 12 [Y = Cl] and 5 [Y = I] kcal/mol, and FSTS_I_ is below the front-side [FICH_3_]^−^ transition state of F^–^ + CH_3_I by ∼8 kcal/mol. Regarding the halogen-bonded complex formation, it should be emphasized that for F^–^ + CH_3_Cl, the front-side [F···ClCH_3_]^−^ complex is very weakly bound (*D*_e_ = 2.7 kcal/mol); on the contrary, the analogous FSMIN_Cl_ is much deeper (*D*_e_ = 12.2 kcal/mol) for the F^–^ + CH_2_ClI system; moreover, a more stable FSMIN_I_ (*D*_e_ = 32.2 kcal/mol) is found compared to the F^–^ + CH_3_I case. For the proton-abstraction channel of F^–^ + CH_2_ClI, following the entrance region, the entire reaction pathway is situated over 15 kcal/mol lower than the F^–^ + CH_3_I → HF + CH_2_I^–^ pathway, and the corresponding HF + CHClI^–^ products are submerged by ∼20 kcal/mol compared to HF + CH_2_I^–^. Furthermore, the relative barrier heights of the transition states along the proton-abstraction route are also less significant for F^–^ + CH_2_ClI, and the relative barrier heights of TS1_Cl_ and TS1_I_ are comparable with the barriers of WaldenTS_Cl_ and WaldenTS_I_. Thus, proton abstraction displays a more enhanced kinetic and thermodynamic character for the reaction studied in the present work. Taken together, these findings suggest that the additional halogenation of CH_3_Y slightly hinders the probability of the traditional back-side attack Walden inversion as it significantly promotes the competing proton-abstraction channel as well as the S_N_2 retention pathways and the halogen-bonded complex formation.

**Figure 5:**
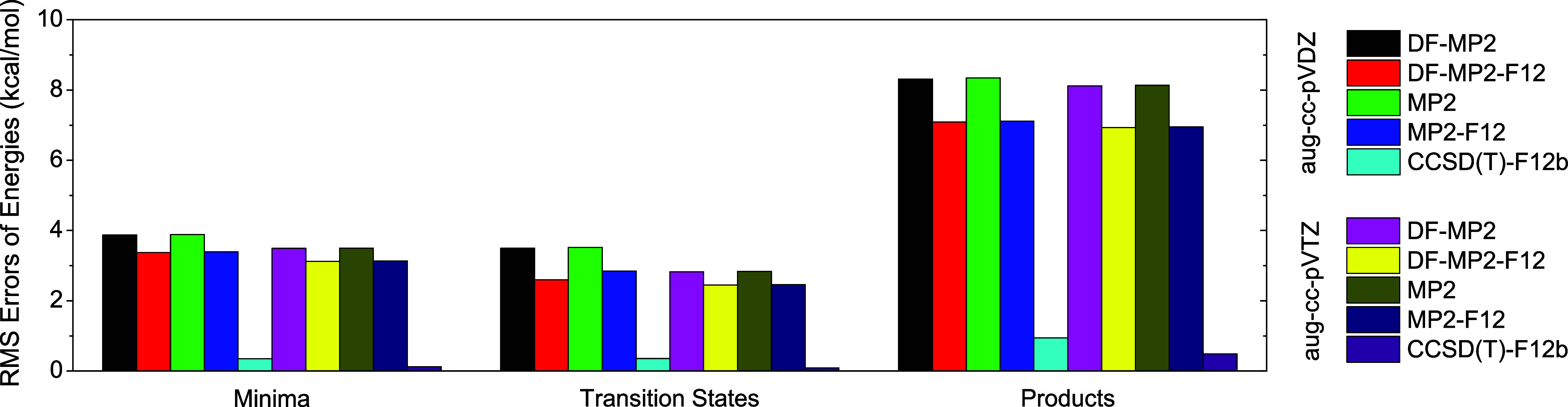
RMS errors of the classical energies of the complexes (minima), transition states, and products obtained at various levels of theory relative to the CCSD(T)-F12b/aug-cc-pVQZ values in the case of the F^–^ + CH_2_ClI S_N_2 and proton-abstraction reactions.

**Figure 6:**
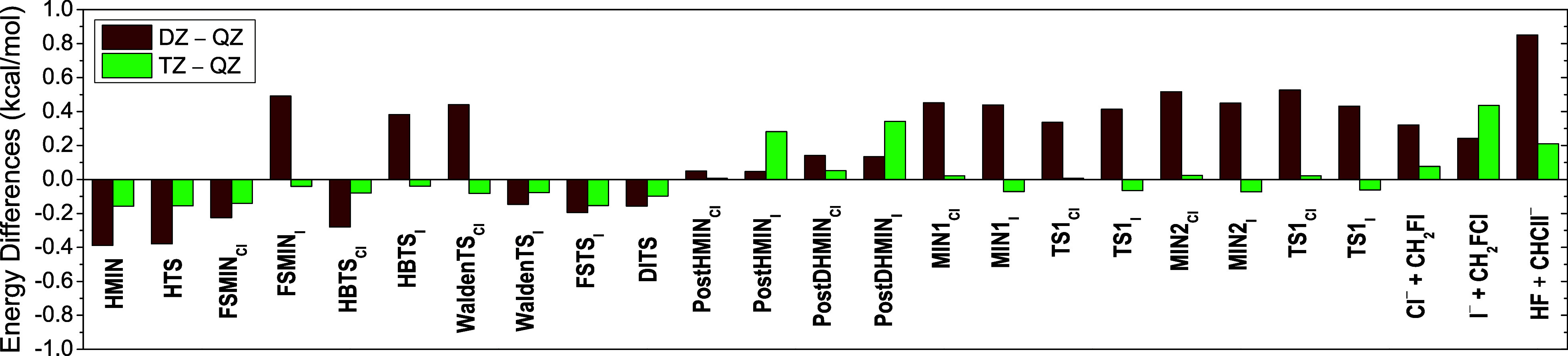
Basis-set convergence of the CCSD(T)-F12b relative energies using the aug-cc-pVDZ (DZ), aug-cc-pVTZ (TZ), and aug-cc-pVQZ (QZ) basis sets for the stationary points of the S_N_2 and proton-abstraction channels of the F^–^ + CH_2_ClI reaction.

**Figure 7:**
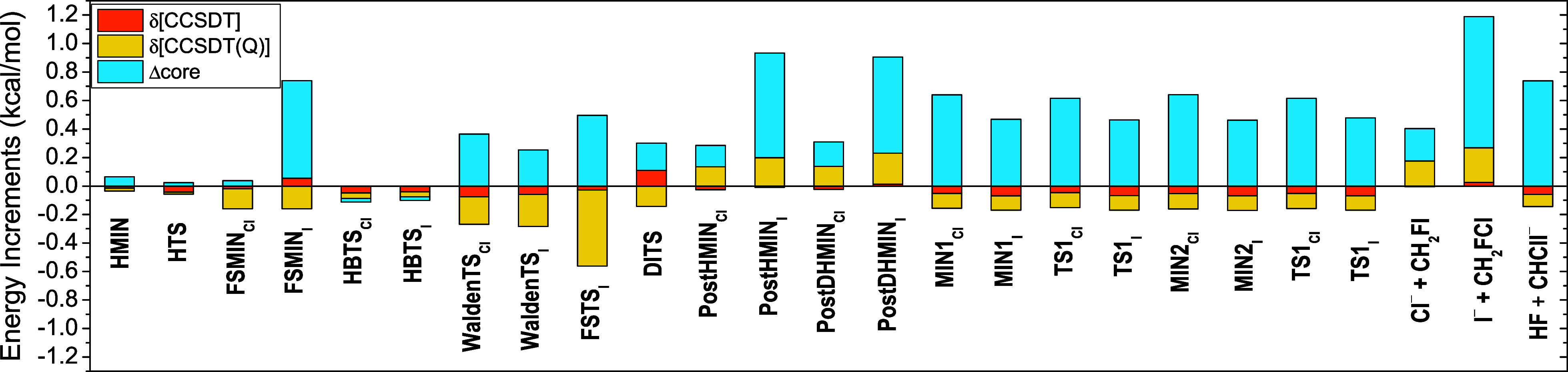
Post-CCSD(T), eqs 2 and 3, and core correlation, eq 4, energy effects for the stationary points of the F^–^ + CH_2_ClI S_N_2 and proton-abstraction reactions.

The classical relative energies obtained by the DF-MP2, DF-MP2-F12, MP2, and MP2-F12 methods with the aug-cc-pVDZ and aug-cc-pVTZ basis sets are summarized in Table 2. The root-mean-square (RMS) deviations of the relative classical energies computed at the formerly mentioned levels of theory and at CCSD(T)-F12b/aug-cc-pVQZ are shown in Figure 5. In case of the complexes and transition states, the RMS values of the MP2 methods are in the range of 2.4–3.9 kcal/mol; however, for the products, higher RMS values are obtained, within 6.9–8.4 kcal/mol. Among the applied MP2 methods, the smallest RMS errors are determined for the explicitly-correlated F12 variants, and the largest differences (even >5 kcal/mol) between the relative energies provided by the non-F12 and F12 methods are obtained at DITS. The density-fitted DF-MP2 and DF-MP2-F12 methods preform nearly identically to the non-DF methods at the corresponding basis set. The most pronounced difference (0.22 kcal/mol) of the RMS values is found to be for the transition states in the case of the calculations performed at the DF-MP2-F12/aug-cc-pVDZ and MP2-F12/aug-cc-pVDZ levels of theory. As demonstrated by the RMS values of the MP2 methods, applying the aug-cc-pVTZ basis set does not substantially improve the accuracy of the relative energies compared to aug-cc-pVDZ. Therefore, the application of these MP2 methods is deemed unsuitable for the current system as they do not provide chemical accuracy for the relative energies of the stationary points. On the contrary, the RMS errors of the CCSD(T)-F12b method drop below 1 kcal/mol, reaching the values of 0.13, 0.09, and 0.49 kcal/mol at the aug-cc-pVTZ basis set for the complexes, transition states, and products, respectively. The deviations of the computed CCSD(T)-F12b energies using the aug-cc-pV*n*Z [*n* = 2 (DZ), 3 (TZ), and 4 (QZ)] basis sets are depicted in Figure 6; also, the post-CCSD(T) and the core correlations, obtained from eqs 2–4, are shown for the stationary points of the F^–^ + CH_2_ClI reaction in Figure 7. As expected, the explicitly-correlated CCSD(T)-F12b method displays a fast basis-set convergence: typically, the DZ versus QZ differences are within ±0.55 kcal/mol, while the deviations of TZ and QZ are within ±0.20 kcal/mol. The largest difference (0.85 kcal/mol) can be identified for the HF + CHClI^–^ products; moreover, in the case of PostHMIN_I_, PostDHMIN_I_, and I^–^ + CH_2_FCl, the basis-set convergence breaks as the obtained TZ–QZ is higher than the corresponding DZ–QZ by 0.23, 0.21, and 0.19 kcal/mol, respectively. As seen in Figure 7, core corrections usually have an opposite sign to the post-CCSD(T) effects, thereby reducing their combined impact and resulting in energy contributions within the 0.3–0.6 kcal/mol range. However, there are instances when their effects are cumulative: significant auxiliary energy contributions of 1.2 and 0.9 kcal/mol are determined for the I^–^ + CH_2_FCl products and the PostDHMIN_I_ complex, signifying the importance of their consideration. The ZPE corrections of the stationary points obtained at the CCSD(T)-F12b/aug-cc-pVTZ level of theory are presented in Table 1. As it can be seen, for the FSMIN_Cl_, FSMIN_I_, PostHMIN_Cl_, PostHMIN_I_, PostDHMIN_Cl_, PostDHMIN_I_ complexes and the HBTS_Cl_ and HBTS_I_ transition states as well as for the Cl^–^ + CH_2_FI and I^–^ + CH_2_FCl products, positive ΔZPEs can be determined, which increase the energy levels of the stationary points and decrease the exothermicity of the S_N_2 channels. In almost every case, the ZPE effects are within ±2.0 kcal/mol, except for the HF + CHClI^–^ products, where a substantial value of −3.15 kcal/mol is observed. Similarly, in the cases of the Cl^–^ + CH_2_FI and I^–^ + CH_2_FCl S_N_2 products, noticeable ZPE corrections of 1.03 and 1.84 kcal/mol can be recognized, demonstrating that the consideration of these ZPE corrections, along with the core and post-CCSD(T) effects, is required in order to achieve accurate reaction enthalpies.

**Table 2:** Classical Relative Energies (kcal/mol) of the Stationary Points, Obtained Using the DF-MP2, DF-MP2-F12, MP2, and MP2-F12 Methods with the aug-cc-pV*n*Z [*n* = 2 and 3] Basis Sets, for the S_N_2 and Proton-Abstraction Channels of the F^–^ + CH_2_ClI Reaction

basis set	aug-cc-pVDZ				aug-cc-pVTZ			
method	DF-MP2	DF-MP2-F12	MP2	MP2-F12	DF-MP2	DF-MP2-F12	MP2	MP2-F12
HMIN	–26.94	–26.91	–26.94	–26.90	–26.51	–26.60	–26.51	–26.59
HTS	–20.61	–20.91	–20.60	–20.90	–20.40	–20.54	–20.40	–20.54
FSMIN_Cl_	–9.84	–11.57	–9.81	–11.54	–10.52	–11.48	–10.51	–11.47
FSMIN_I_	–30.35	–31.59	–30.34	–31.57	–31.07	–31.98	–31.06	–31.97
HBTS_Cl_	–3.73	–4.67	–3.71	–4.65	–4.13	–4.42	–4.12	–4.41
HBTS_I_	–6.13	–6.92	–6.13	–6.92	–7.03	–7.22	–7.03	–7.22
WaldenTS_Cl_	–11.93	–10.50	–11.91	–10.48	–10.86	–10.72	–10.85	–10.71
WaldenTS_I_	–16.21	–16.07	–16.19	–16.06	–15.21	–15.75	–15.20	–15.74
FSTS_I_	12.72	13.09	12.74	13.11	13.61	13.35	13.63	13.37
DITS	9.45	4.24	9.46	4.26	5.75	4.24	5.77	4.25
PostHMIN_Cl_	–46.06	–45.33	–46.05	–45.32	–45.47	–45.08	–45.46	–45.08
PostHMIN_I_	–58.39	–a	–58.37	–a	–a	–a	–a	–a
PostDHMIN_Cl_	–45.62	–45.04	–45.61	–45.03	–45.01	–44.86	–45.00	–44.85
PostDHMIN_I_	–58.41	–60.31	–58.39	–60.29	–58.54	–59.63	–58.54	–59.62
MIN1_Cl_	–8.24	–8.74	–8.22	–8.72	–8.98	–9.42	–8.97	–9.41
MIN1_I_	–6.49	–6.93	–6.47	–6.91	–7.31	–7.74	–7.30	–7.73
TS1_Cl_	–8.19	–8.64	–8.16	–8.62	–8.81	–9.25	–8.80	–9.23
TS1_I_	–6.18	–6.60	–6.15	–6.58	–6.92	–7.38	–6.90	–7.36
MIN2_Cl_	–8.03	–8.61	–8.01	–8.59	–8.89	–9.34	–8.87	–9.33
MIN2_I_	–6.61	–7.11	–6.59	–7.09	–7.48	–7.92	–7.47	–7.91
TS2_Cl_	–6.81	–7.38	–6.79	–7.36	–7.66	–8.13	–7.65	–8.12
TS2_I_	–5.80	–a	–5.78	–6.31	–6.64	–7.12	–6.63	–7.11
Cl^–^ + CH_2_FI	–31.40	–30.73	–31.39	–30.73	–30.83	–30.68	–30.83	–30.68
I^–^ + CH_2_FCl	–47.62	–49.89	–47.59	–49.86	–47.91	–49.16	–47.90	–49.15
HF + CHClI^–^	5.54	5.32	5.56	5.34	5.15	4.36	5.16	4.37

a Geometry optimization does not converge.

## 4 Summary and Conclusions

In this work, we investigated the potential energy surfaces of the S_N_2 and proton-abstraction channels of the F^–^ + CH_2_ClI reaction using the explicitly-correlated CCSD(T)-F12b method with the aug-cc-pV*n*Z [*n* = 2 and 3] basis sets. Since there are two possible halide ions (Cl^–^ and I^–^) that can act as a leaving group in the S_N_2 process, we have characterized two distinct S_N_2 pathways leading to the Cl^–^ + CH_2_FI and I^–^ + CH_2_FCl products. It was verified that the formation of I^–^ + CH_2_FCl is thermodynamically more favored than that of Cl^–^ + CH_2_FI. Within each S_N_2 channel, four distinct pathways have been considered: back-side attack, front-side attack, double inversion,^17^ and halogen-bonded complex formation.^34,60^ In the entrance region of the I^–^ + CH_2_FCl S_N_2 channel, stable front-side complex formation has been unveiled as the corresponding [F···IClCH_2_]^−^ complex is located below the reactant asymptote by an enormous classical (adiabatic) energy of 32.2 (32.1) kcal/mol. Regarding the S_N_2 retention pathways, the barrier height of the front-side attack path of the I^–^ + CH_2_FCl S_N_2 channel is situated above that of double inversion by 7.7 (9.3) kcal/mol; however, no front-side transition state can be identified in the case of the Cl^–^ + CH_2_FI channel. Moreover, the comparison with the conventional F^–^ + CH_3_Y [Y = Cl and I] S_N_2 reactions^17,32^ uncovered that the additional halogenation of CH_3_Y causes a somewhat less preferred back-side attack Walden-inversion pathway since it enhances the prevalence of the proton-abstraction and S_N_2 retention channels as well as of the halogen-bonded complex formation. However, it should be emphasized that these findings, derived from the energy profiles of the possible pathways, await validation through subsequent theoretical and experimental examinations on the dynamics of the title reaction. The basis-set convergence of the CCSD(T)-F12b methods, as well as the ZPE contributions and the auxiliary energy corrections of post-CCSD(T) and core correlations, have also been investigated. Furthermore, the stationary points have been characterized by utilizing the MP2, DF-MP2, MP2-F12, and DF-MP2-F12 methods in order to test the performance of different ab initio levels of theory for the F^–^ + CH_2_ClI system.

Overall, the present study highlights the incomplete understanding of even basic S_N_2 reactions involving additionally halogenated substrate molecules; therefore, we expect that this work will pave the way for further comprehensive experimental and theoretical examinations of such reactions.

## Supporting Information Available

Benchmark Cartesian coordinates (Å) and energies (*E*_h_) of the stationary points (PDF)
