# Supplementary material for: Benchmark Ab Initio Mapping of the F– + CH2ClI SN2 and Proton-Abstraction Reactions
Source: J Phys Chem A. 2024 Dec 2;128(49):10568–78. doi: 10.1021/acs.jpca.4c06716 (PMC11647900; doi:10.1021/acs.jpca.4c06716)
Supplement: Supplementary file 1 — jp4c06716_si_001.pdf [file jp4c06716_si_001.pdf]

Benchmark ab Initio Mapping of the F^- + CH_2ClI S_N2 and Proton-Abstraction Reactions

Domonkos A. Tasi[a]*, Erik M. Orján[a] and Gábor Czakó[a]*

[a] *MTA-SZTE Lendület "Momentum" Computational Reaction Dynamics Research Group, Interdisciplinary Excellence Centre and Department of Physical Chemistry and Materials Science, Institute of Chemistry, University of Szeged, Szeged H-6720, Hungary*

* Email: dtasi@chem.u-szeged.hu and gczako@chem.u-szeged.hu

6

HMIN CCSD(T)-F12B/AVTZ, I=AVTZ-PP BENCHMARK ENERGY=-894.818659156431

C	-0.5557539598	-0.0615697617	-1.0982269803
I	0.0206892831	-0.0215060561	0.9965217383
Cl	0.1009171792	1.3623625908	-1.9591819668
H	-1.6335214727	0.0690173237	-1.0697806861
H	-0.1857906031	-1.0778234392	-1.5733392375
F	0.1213533433	-2.3062083607	-2.1659821083

6

HTS CCSD(T)-F12B/AVTZ, I=AVTZ-PP BENCHMARK ENERGY=-894.809354232902

C	0.0000217877	-0.2396059000	-1.1823019349
I	0.0000042800	0.0841395589	0.9452452617
Cl	0.0000019776	1.3078428608	-2.0798595323
H	0.8701507424	-0.8367060087	-1.4191426749
H	-0.8710521505	-0.8358607998	-1.4176665321
F	0.0000017688	-2.7623881792	-1.5348398504

6

FSMIN-Cl CCSD(T)-F12B/AVTZ, I=AVTZ-PP BENCHMARK ENERGY=-894.794841676115

C	0.0000000000	-1.1552774284	0.5268424378
Cl	0.0000000000	-0.2943511888	2.0648781272
I	0.0000000000	0.1244719669	-1.2555644923
H	-0.8964303506	-1.7546844958	0.4020501789
H	0.8964303506	-1.7546844958	0.4020501789
F	0.0000000000	0.6344079829	4.1578968738

6

FSMIN-I CCSD(T)-F12B/AVTZ, I=AVTZ-PP BENCHMARK ENERGY=-894.826782957539

C	-0.8514525836	0.0000000000	-1.5279279326
Cl	0.4297671471	0.0000000000	-2.8090450644
I	-0.1028079022	0.0000000000	0.5544760500
H	-1.4470429483	-0.8910252001	-1.7104065116
H	-1.4470429483	0.8910252001	-1.7104065116
F	0.5765855798	0.0000000000	2.6856460743

6

HBTS-Cl CCSD(T)-F12B/AVTZ, I=AVTZ-PP BENCHMARK ENERGY=-894.783203185137

C	-0.6995477863	0.0000000000	-0.7544598219
Cl	0.5467251714	0.0000000000	-1.9519908572
I	-0.0126077380	0.0000000000	1.3260439642
H	-1.3009591941	-0.8970847402	-0.8380640397
H	-1.3009591941	0.8970847402	-0.8380640397
F	-0.3557252358	0.0000000000	-4.6491778480

6

HBTS-I CCSD(T)-F12B/AVTZ, I=AVTZ-PP BENCHMARK ENERGY=-894.787729049815

C	-0.1725598084	-0.9730477144	1.1285475376
Cl	0.0625081070	-0.3139740857	2.7824822135
I	-0.0061651193	0.4037403118	-0.4753107983
H	-1.1675837796	-1.3989801458	1.0919333316
H	0.5836961429	-1.7326871911	0.9729297436
F	0.0646068289	-1.3296745855	-2.8404533525

6

Waldents-Cl CCSD(T)-F12B/AVTZ, I=AVTZ-PP BENCHMARK ENERGY=-894.796103208026

C	0.0000000000	-0.9721841295	-0.8523352699
I	0.0000000000	0.3903119005	0.7516622758
Cl	0.0000000000	0.3440145123	-2.5558472545
H	-0.9425445712	-1.4010276696	-1.1156853257
H	0.9425445712	-1.4010276696	-1.1156853257
F	0.0000000000	-2.4858840572	0.4057676635

6

WaldenTS-I CCSD(T)-F12B/AVTZ, I=AVTZ-PP BENCHMARK ENERGY=-894.804128463238

C	-0.4104873963	0.0000000000	-1.2689261713
Cl	1.1775676126	0.0000000000	-1.9575567061
I	-0.0668030004	0.0000000000	1.1563185784
H	-0.9340999762	-0.9318266597	-1.3095878561
H	-0.9340999762	0.9318266597	-1.3095878561
F	-1.3926036576	0.0000000000	-3.1297772558

6

FSTS-I CCSD(T)-F12B/AVTZ, I=AVTZ-PP BENCHMARK ENERGY=-894.75681092273

C	-0.7990925877	-0.0683862717	1.3463509645
Cl	0.1904742092	-0.5201091548	2.7314935248
F	0.0967099278	1.6387606067	1.2485192209
I	0.0297662744	-0.0904378399	-1.0977018550
H	-1.5903434315	0.6233142603	1.5847844702
H	-1.1576536822	-1.0161618480	0.9687011561

6

DITS CCSD(T)-F12B/AVTZ, I=AVTZ-PP BENCHMARK ENERGY=-894.769158176286

F	-0.0985135442	-2.2686165450	1.8590676361
H	0.1627780108	-1.4118265889	1.4985725563
C	0.8043234658	0.2706279429	0.9096485043
Cl	-0.1532385613	1.0911432890	2.1372601562
I	-0.0326137727	0.0233604936	-0.9831407076
H	1.6056818671	-0.3732961911	1.2282525462

6

PostHMIN-Cl CCSD(T)-F12B/AVTZ, I=AVTZ-PP BENCHMARK ENERGY=-894.852786741971

C	-0.3683558805	0.9656998290	-0.6138612874
I	0.0122061576	-0.2872330098	1.1177908969
F	0.1626525362	2.2080466681	-0.3892326906
H	-1.4488994480	1.0437091413	-0.6811438039
H	0.0724702676	0.4733009756	-1.4869466351
Cl	0.0330723709	-0.5253720776	-3.5229884177

6

PostHMIN-I MP2/AVDZ, I=AVDZ-PP BENCHMARK ENERGY=-894.874544491711

C	-0.4351705514	0.2615251129	2.1422488413
Cl	0.9021393859	-0.0402585270	3.3052210538
F	-1.6353067241	-0.1034249271	2.7491185497
H	-0.2665028499	-0.3307675207	1.2346555287
H	-0.4580526863	1.3368391637	1.9363704360
I	0.0397288888	-0.0060126991	-1.5628619339

6

PostDHMIN-Cl CCSD(T)-F12B/AVTZ, I=AVTZ-PP BENCHMARK ENERGY=-894.852398044611

C	0.0000000000	0.9560672013	-0.6736972940
I	0.0000000000	-0.2981956497	1.0984558409
F	0.0000000000	2.2704250931	-0.2848488335
H	-0.8902888861	0.7084495011	-1.2381529098
H	0.8902888861	0.7084495011	-1.2381529098
Cl	0.0000000000	-0.5134483691	-3.4806803108

6

PostDHMIN-I CCSD(T)-F12B/AVTZ, I=AVTZ-PP BENCHMARK ENERGY=-894.875535240344

C	-0.4211957874	0.0000000000	2.0859809859
Cl	0.8874412711	0.0000000000	3.3026233967
F	-1.6217732686	0.0000000000	2.7430467259
H	-0.3253285222	-0.8924462121	1.4786965292
H	-0.3253285222	0.8924462121	1.4786965292
I	0.0398994260	0.0000000000	-1.5542049969

6

TS1-Cl CCSD(T)-F12B/AVTZ, I=AVTZ-PP BENCHMARK ENERGY=-894.794853597443

C	-0.9331866116	0.0898719392	-0.8226674587
Cl	0.1060225757	-1.1037604021	-1.8676898112
I	0.0504660402	0.1209335990	1.2179652521
H	-1.7411100373	-0.6106374843	-0.5488840348
H	0.0607685829	0.6190642518	-3.1991240440
F	0.1441677156	1.1946554265	-3.9315059546

6

TS1-I CCSD(T)-F12B/AVTZ, I=AVTZ-PP BENCHMARK ENERGY=-894.792505976212

C	-0.9091087659	-0.7756239058	1.0653167696
I	0.6183704660	0.0116612007	-0.4802733331
Cl	-0.6038292131	0.1996177962	2.6191324308
H	-0.3244158267	-1.6805717445	1.3109343585
H	-1.6221517244	0.0627916909	-1.7206289544
F	-2.3257568314	0.1257855672	-2.3312167220

6

MIN1-Cl CCSD(T)-F12B/AVTZ, I=AVTZ-PP BENCHMARK ENERGY=-894.795215818338

C	-1.0517426137	-0.0048589600	-0.7085697991
Cl	0.1389749687	-0.8954714767	-1.8910721502
I	0.0564323921	0.1122890578	1.2666383817
H	-1.6655493385	-0.8825066216	-0.4378356747
H	0.0800766390	0.4406064094	-3.4850332067
F	0.1127403722	0.9474918673	-4.2758552509

6

MIN1-I CCSD(T)-F12B/AVTZ, I=AVTZ-PP BENCHMARK ENERGY=-894.793108400658

C	0.9029725282	-0.8211260685	-1.2343364185
I	-0.4957563212	0.0013342186	0.4078023732
Cl	0.5834319762	0.2417207162	-2.7295205023
H	0.2412070131	-1.6703994480	-1.4855341659
H	1.0718970392	0.0891922814	2.3202293021
F	1.5822702184	0.1430258672	3.1056115182

6

TS2-Cl CCSD(T)-F12B/AVTZ, I=AVTZ-PP BENCHMARK ENERGY=-894.793326892524

C	0.2411730284	-1.6556310184	0.2769971414
Cl	-0.0525978459	-1.0435242657	2.0574419022
I	-0.0078742982	0.1692451596	-1.0385856675
H	-0.7732427677	-2.0578424040	0.1021604880
H	0.0182497349	1.0585115795	2.4145947833
F	0.0383348443	1.9165290255	2.7894918765

6

TS2-I CCSD(T)-F12B/AVTZ, I=AVTZ-PP BENCHMARK ENERGY=-894.79217595516

C 0.4147542061 -1.0672794336 -1.4823308743

I -0.0236081373 -0.4574500684 0.7075153733

Cl -0.0823845612 0.3968815714 -2.5124455180

H -0.5088606528 -1.6676135742 -1.5797770312

H 0.0538497805 2.0503244652 0.7673319042

F 0.0733635790 2.9694909212 0.9426782644

6

MIN2-Cl CCSD(T)-F12B/AVTZ, I=AVTZ-PP BENCHMARK ENERGY=-894.795122281042

C 0.9237826159 -1.0023074160 0.5243290463

Cl 1.0137409465 0.2791731223 1.9239146954

I -0.1600002242 0.0404949893 -1.1736501977

H 0.0462822095 -1.5638564988 0.8923287631

H -0.6558418019 0.0395406523 3.1089063703

F -1.3746639034 -0.0769235302 3.7057472141

6

MIN2-I CCSD(T)-F12B/AVTZ, I=AVTZ-PP BENCHMARK ENERGY=-894.793357376946

C 0.0977129996 -1.0195511073 -1.5048869938

I 0.5636148257 0.0542498741 0.4834742553

Cl -0.7977297335 0.2284458574 -2.5535368082

H -0.7745691785 -1.5676500512 -1.1042253739

H -1.4774778297 -0.0203224487 1.8706250515

F -2.2184788496 -0.0598620632 2.4464034945

1

F^- CCSD(T)-F12B/aug-cc-pVTZ BENCHMARK ENERGY=-99.845471575799

F 0.0000000000 0.0000000000 0.0000000000

5

CH_2ClI CCSD(T)-F12B/AVTZ, I=AVTZ-PP BENCHMARK ENERGY=-794.929929492327

C 0.0000000000 -0.9089656411 1.1102832090

Cl 0.0000000000 0.2330240387 2.4557370721

I 0.0000000000 0.0449681509 -0.8095969783

H 0.8972094580 -1.5132429012 1.1624552683

H -0.8972094580 -1.5132429012 1.1624552683

5

CH_2FCl CCSD(T)-F12B/AVTZ BENCHMARK ENERGY=-599.166295310957

C 0.0000000000 -0.6318714296 -0.5648959807

Cl 0.0000000000 0.1031093486 1.0435745399

F 0.0000000000 0.3372411308 -1.5200525717

H 0.9032222246 -1.2268443095 -0.6619001128

H -0.9032222246 -1.2268443095 -0.6619001128

1

I^- CCSD(T)-F12B/aug-cc-pVTZ BENCHMARK ENERGY=-295.692408499529

I 0.0000000000 0.0000000000 0.0000000000

5

CH_2FI CCSD(T)-F12B/AVTZ, I=AVTZ-PP BENCHMARK ENERGY=-434.726353601521

C 0.0000000000 -0.6620459279 -1.5114849949

I 0.0000000000 0.0235697887 0.5244779433

F 0.0000000000 0.3933785835 -2.3727870496

H 0.9040154309 -1.2465348578 -1.6496380314

H -0.9040154309 -1.2465348578 -1.6496380314

1

Cl^- CCSD(T)-F12B/aug-cc-pVTZ BENCHMARK ENERGY=-460.103473604266

Cl 0.0000000000 0.0000000000 0.0000000000

4

$CHClI^-$ CCSD(T)-F12B/AVTZ, I=AVTZ-PP BENCHMARK ENERGY=-794.328213896409

C -0.1004285401 -1.0766013440 1.1791104847

Cl 0.0041016235 0.2427310764 2.5055518819

I 0.0007149205 0.0450392858 -0.8208336189

H 0.9624631087 -1.3792633345 1.1673232891

2

HF CCSD(T)-F12B/AVTZ BENCHMARK ENERGY=-100.445381813911

F 0.0000000000 0.0000000000 -0.0462297626

H 0.0000000000 0.0000000000 0.8713729592
